# Siberian Sturgeon (*Acipenser baerii*, Brandt, 1869) the Relationship Between Genetic Resources and Acclimatization: Ecology, Conservation, and the Nutritional Significance of Meat and Caviar

**DOI:** 10.3390/life16091437

**Published:** 2026-08-28

**Authors:** Cătălina Teodora Cîrmaciu (Florea), Cătălin Emilian Nistor, Gabriel-Vasile Hoha, Benone Păsărin

**Affiliations:** Department of Animal Resources and Technologies, Faculty of Food and Animal Sciences, “Ion Ionescu de la Brad” University of Life Sciences, 8 Mihail Sadoveanu Alley, 700489 Iasi, Romania; catalina.florea@iuls.ro (C.T.C.); gabriel.hoha@iuls.ro (G.-V.H.); benone.pasarin@iuls.ro (B.P.)

**Keywords:** sturgeon meat, caviar, nutritional quality, no-kill technology, sustainable aquaculture, conservation, *Acipenser baerii*, reproductive biology, polyunsaturated fatty acids

## Abstract

The Siberian sturgeon (*Acipenser baerii*, Brandt, 1869) is a potamodromous freshwater species with major ecological and commercial importance. Natural populations across the main Siberian River basins have declined so severely that the species is now classified as Critically Endangered on the IUCN Red List. This narrative analysis synthesizes current scientific knowledge regarding the ecology, reproductive biology, conservation status, and nutritional value of products derived from *A. baerii*, with the aim of providing an integrated perspective on this species, which has recently been introduced into Romanian aquaculture and occupies the interface between biodiversity and human consumption needs. The species’ remarkable ecological and morphological plasticity, which underlies its wide natural distribution in the Ob, Irtysh, Yenisei, and Lena River basins, has also made it the dominant species in global sturgeon aquaculture, commercially farmed in Europe, Asia, and South America. Reproductive management in farming systems relies on hormonal induction, non-invasive maturity assessment, and controlled hatchery practices, while emerging no-kill technologies enable repeated caviar harvesting without sacrificing the female, improving both ethical standards and economic efficiency. From a nutritional standpoint, *A. baerii* yields two high-value products: caviar, a rich source of the long-chain polyunsaturated fatty acids EPA and DHA, whose dietary intake has been associated with cardiovascular and neurodevelopmental benefits in the broader nutrition literature, and meat, characterized by high-biological-value protein, a favorable amino acid profile, and significant omega-3 content. By-products such as skin, swim bladders, glands, and cartilage contribute to the higher-value utilization of the species within the circular bioeconomy. Still, notable gaps remain in genetics and sex determination methods, in early life behavioral ecology, and in cryopreservation protocols. Future work should also look at more sustainable feed formulations and new value-added products.

## 1. Introduction

Sturgeons are among the oldest and most evolutionarily distinct vertebrate lineages alive today. The order *Acipenseriformes* is characterized by the retention of several ancestral morphological features that distinguish it from most other extant ray-finned fishes. These include a predominantly cartilaginous endoskeleton, a heterocercal caudal fin, rows of bony scutes instead of true scales, and the persistence of the notochord throughout adulthood [1,2,3,4,5,6]. According to the current taxonomic classification of Eschmeyer’s Catalog of Fishes (California Academy of Sciences), the family *Acipenseridae* comprises 27 extant species, traditionally classified into four genera (*Acipenser*, *Huso*, *Scaphirhynchus*, and *Pseudoscaphirhynchus*). It represents one of the oldest surviving lineages of *Actinopterygii*, with an evolutionary history extending back more than 200 million years. Despite their long evolutionary history, approximately 85% of *Acipenseridae* species are currently classified as threatened according to the IUCN Red List, making sturgeons the most threatened vertebrate group worldwide [7,8,9,10].

Interest in sturgeon biology has increased substantially over approximately the last thirty years. This has been driven by conservation urgency, by efforts to rebuild depleted wild stocks, and by the fast growth of sturgeon aquaculture worldwide [7,10,11]. Early work on *Acipenser baerii* was largely restricted to the Ob and Irtysh basins, mostly because Siberian habitats are remote and the wild populations there were already sparse. It was not until 1961 that anything like systematic research on the eastern populations got underway, with long-term studies in the Lena River basin providing the first real picture of the species’ ecology and life history [12,13]. Since then, research has expanded substantially. Asian studies, particularly those conducted in China, have primarily focused on aquaculture production, reproductive physiology, nutrition, and environmental adaptation, reflecting the dominant role of these countries in global sturgeon farming. In contrast, European research, especially from Russia, Italy, France, Romania, Poland, and Germany, has contributed extensively to genetics and genomics, reproductive biotechnology, conservation, fish welfare, and product quality, including meat and caviar characterization [14,15,16].

From a nutritional point of view, *A. baerii* is an opportunistic benthic feeder, consuming primarily aquatic insect larvae (particularly *Chironomidae*, as well as larvae of *Ephemeroptera*), together with oligochaetes, freshwater mollusks, crustaceans (e.g., *Daphnia* spp.), and occasionally small fishes, depending on habitat and ontogenetic stage [12,17,18]. This dietary flexibility carries over well into captivity. The species can grow efficiently on diets high in plant-derived protein without any real loss in performance, so long as essential amino acid needs are covered [19,20,21]. This has made it an attractive candidate for sustainable feed development, meaning feeds that cut back on fishmeal without sacrificing growth or welfare in intensive systems [8,22,23]. Despite being critically threatened throughout its natural range, *A. baerii* adapts exceptionally well to captive conditions, making it one of the most important species used in commercial sturgeon aquaculture worldwide [3,4,11,24,25]. Its relatively rapid growth, broad environmental tolerance, reliable artificial reproduction, and excellent performance in recirculating aquaculture systems (RAS) have established it as a valuable model species for developing intensive farming protocols. However, these characteristics should not be generalized to all *Acipenseridae*, as sturgeon species differ considerably in salinity tolerance, migratory behavior, reproductive biology, and other life history traits [8,11,16].

Unlike the four native sturgeon species occurring in the Romanian sector of the Danube basin (*Huso huso*, *Acipenser gueldenstaedtii*, *A. stellatus*, and *A. ruthenus*), *Acipenser baerii* is a non-native species that is cultivated in Romania exclusively under controlled aquaculture conditions. Its suitability for intensive production, relatively rapid growth, adaptability to captive conditions, and high commercial value for both meat and caviar have contributed to its inclusion among the sturgeon species cultivated in Romanian aquaculture [26,27].

This narrative review synthesizes current knowledge on the ecology, reproductive biology, aquaculture production systems, meat and caviar quality, and conservation of *A. baerii*, while identifying current knowledge gaps and highlighting future research priorities for sustainable sturgeon production and management. It is intended for researchers, aquaculture professionals, fish nutritionists, conservation biologists, fisheries managers, and policymakers interested in sturgeon biology, sustainable aquaculture, and the conservation of endangered *Acipenseridae* species. By integrating knowledge across these interconnected areas, the review aims to support future research and contribute to evidence-based approaches to sustainable production and conservation management.

## 2. Material and Methods

A narrative analysis was conducted by systematically searching major international scientific databases and authoritative institutional sources, including Google Scholar, ScienceDirect, Web of Science, Scopus, PubMed and ResearchGate, as well as technical reports and datasets published by the Food and Agriculture Organization of the United Nations (FAO) and the International Union for Conservation of Nature (IUCN). The searches combined predefined keywords such as *Acipenser baerii*, Siberian sturgeon, sturgeon aquaculture, caviar production, sturgeon meat, reproductive biology, conservation, polyunsaturated fatty acids, no-kill technology, and sustainable aquaculture, using connectors to refine the results. Specifically, the following Boolean query combination was applied across all databases: (“*Acipenser baerii*” OR “Siberian sturgeon”) AND (“aquaculture” OR “reproduction” OR “caviar” OR “meat” OR “conservation” OR “nutrition” OR “no-kill” OR “welfare” OR “sustainable aquaculture”). The specific syntax was adapted to the requirements of each database (e.g., topic field [TS] in Web of Science, title/abstract/keywords [TITLE-ABS-KEY] in Scopus, and advanced search in PubMed). Duplicate records were first removed. The remaining titles and abstracts were then checked against the scope of this analysis, and anything unrelated to the ecology, reproduction, conservation, or nutritional value of *A. baerii* was removed at this stage. A study was excluded if it was only a conference abstract without a full paper behind it or if the methodology was not reported clearly enough to assess its quality. The year of publication was not restricted; the fundamental studies on sturgeon biology date back to 1993, and these remain relevant alongside the most recent works available at the time of writing, so both were retained. After this process, 185 studies were included in the final analysis.

Although a structured literature search and screening procedure were employed to ensure comprehensiveness and reproducibility, these steps do not transform this review into a systematic review or meta-analysis. This is a narrative analysis, and the structured elements of the search strategy serve to support transparency rather than to claim the methodological rigor of a systematic review.

**Figure 1:**
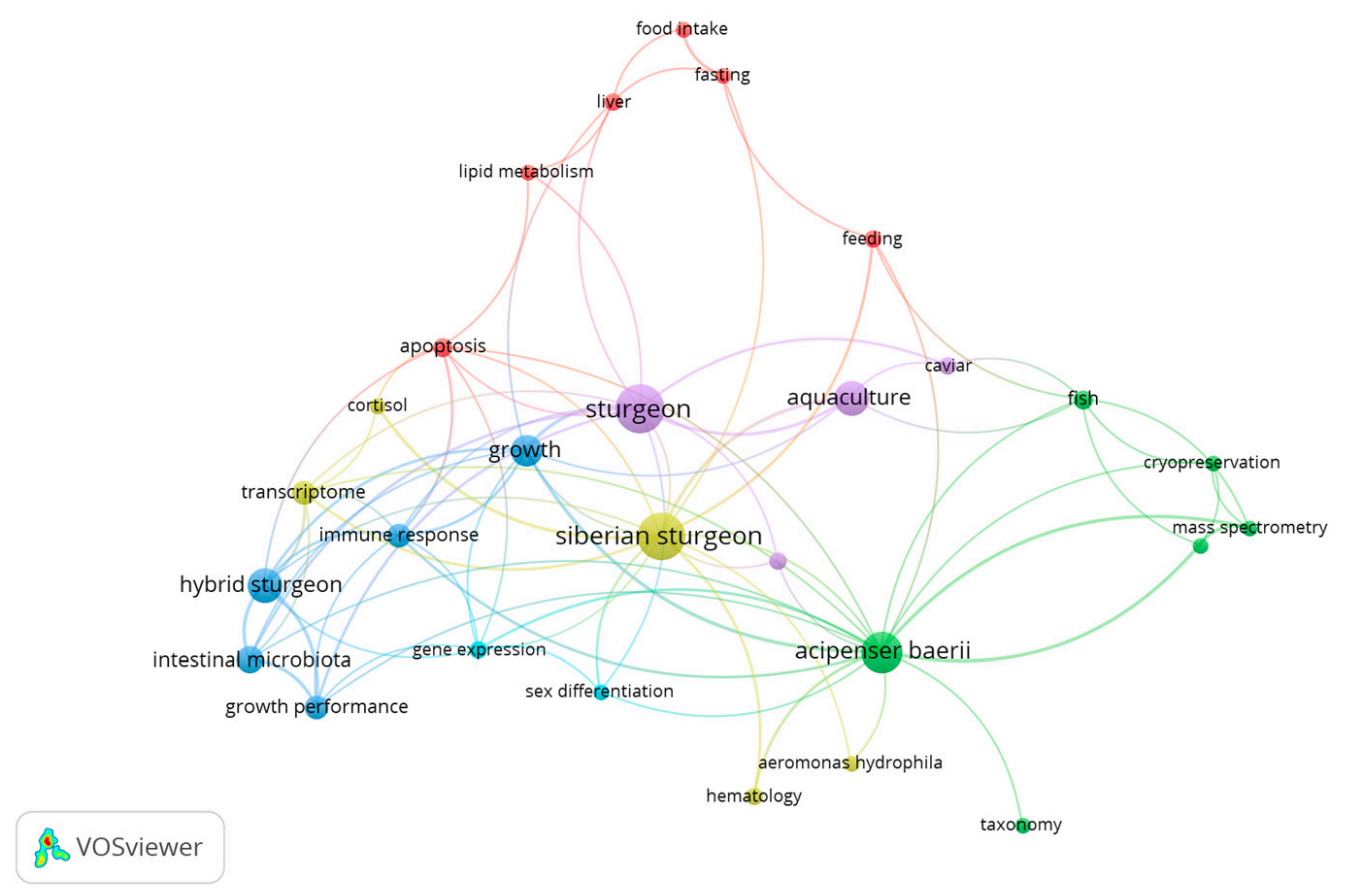
Keyword co-occurrence network derived from Web of Science data (2023–2026) on *Acipenser baerii* research, generated using VOSviewer (v.1.6.20). Node size reflects how often a keyword appears, and thicker connecting lines mean two keywords occur together more often. Four clusters stand out. One group is feeding, fasting, and lipid metabolism. Another centers on growth, immune response, and gut microbiota. A third links taxonomy with cryopreservation and analytical methods such as mass spectrometry. The smallest cluster covers aquaculture, where caviar shows up only as a minor, loosely connected keyword. The VOSviewer co-occurrence map identifies five thematic clusters, each represented by a distinct color: the **red cluster** groups terms related to metabolic physiology and feeding response (food intake, fasting, liver, lipid metabolism, feeding, apoptosis); the **purple cluster** reflects general aquaculture and production aspects (sturgeon, aquaculture, caviar); the **blue cluster** covers growth, stress, and immune-related topics (growth, cortisol, transcriptome, immune response, hybrid sturgeon, intestinal microbiota, growth performance); the **yellow cluster** relates to genetics, reproduction, and pathology (Siberian sturgeon, gene expression, sex differentiation, *Aeromonas hydrophila*, hematology); and the **green cluster** centers on the target species along with conservation biotechnology and analytical methods (*Acipenser baerii*, fish, cryopreservation, mass spectrometry, taxonomy).

A bibliometric analysis was conducted using Web of Science Core Collection records published between 2023 and 2026 to identify recent research trends related to *Acipenser baerii*, the results of which are visualized in Figure 1. This four-year window was selected to capture recent research activity and emerging thematic clusters in *A. baerii* research, complementing the broader historical scope of the narrative review, which encompasses studies dating back to 1993, with a focused snapshot of current trends in the field. The analysis was performed using VOSviewer (version 1.6.20; Leiden University, Leiden, The Netherlands).

Author keyword co-occurrence analysis was conducted using a minimum occurrence threshold of 3. Of the 549 keywords identified in the retrieved records, 31 met the minimum occurrence threshold and were subsequently included in the co-occurrence network. Co-occurrence frequencies were normalized using the association strength method, and clustering was performed using VOSviewer’s default resolution parameter (resolution = 1.0).

## 3. Ecology of the Siberian Sturgeon

The Siberian sturgeon (*Acipenser baerii* Brandt, 1869) is a potamodromous freshwater species characterized by remarkable ecological and morphological plasticity, capable of surviving in habitats with highly variable thermal and trophic conditions [4,7,8,18,28]. Its native range extends across northeastern Eurasia, from the Ob River basin in western Siberia to the Kolyma River basin in northeastern Siberia, encompassing the major Siberian rivers draining into the Arctic Ocean and the Lake Baikal drainage basin [4,7,8,26]. This extensive distribution reflects the remarkable environmental adaptability of the species and has contributed to its successful establishment in commercial aquaculture under diverse production conditions [11]. The Ob River represents one of the principal habitats of the species and is among the most extensively studied river systems within its native range [4,29]. Beyond the major Siberian River systems, *A. baerii* has also been documented in the Angara River and its tributaries based on historical records published between the 1940s and early 1960s, as summarized by Ruban [12].

The Siberian sturgeon was introduced into Romanian aquaculture in the early 2000s, alongside the expansion of intensive sturgeon farming technologies, and has since become one of the principal non-native sturgeon species cultivated because of its rapid growth, adaptability to intensive production systems, and high commercial value for both meat and caviar production [26,27]. Delayed sexual maturation is a common trait throughout the genus *Acipenser*, but *A. baerii* exhibits considerable plasticity in the relationship between growth rate and age at first reproduction, largely influenced by food availability and water temperature [29,30].

The migratory behavior of *A. baerii* differs substantially from that of anadromous sturgeons and is closely adapted to local ecological conditions [30,31]. In the Ob River, both juveniles and adults migrate upstream from the Gulf of Ob for feeding, whereas only mature individuals continue towards the spawning grounds, with spawning migrations occurring between late May and early June at water temperatures of 8–11 °C and ceasing at 18–19 °C [30]. The Lake Baikal population is exclusively potamodromous and undertakes extensive spawning migrations because feeding and spawning habitats are spatially separated. Individuals may migrate up to approximately 1000 km in the Selenga River, more than 300 km in the Barguzin River, and 100–150 km in the Upper Angara River before returning to Lake Baikal, where the principal feeding grounds are located [30,31].

The Siberian sturgeon (*Acipenser baerii*) is classified as Critically Endangered on the IUCN Red List, a classification that reflects the dramatic decline in wild populations throughout its Siberian range [32]. The decline is associated with multiple factors, including overfishing and poaching driven by international demand for caviar, despite international bans and protective regulations in the Ob and Yenisei River basins [33,34]. Furthermore, habitat fragmentation caused by the construction of hydroelectric dams has blocked migration routes and resulted in the loss of access to up to 40% of spawning grounds of the nominal subspecies in the Ob River basin [30,35]. In contrast, severe reproductive abnormalities have been documented in populations of the east Siberian subspecies: in the Kolyma and Indigirka River stocks, 80–100% of females exhibited abnormal gametogenesis during 1987–1989, apparently associated with high levels of water pollution [30,36].

Legally, the species is protected under CITES (Appendix II, 1997–1998) and various regional restrictions, but enforcement remains inconsistent, and poaching continues to threaten wild populations [37]. Integrated strategies are therefore needed, combining in situ conservation with living gene banks and rigorous genetic monitoring, such as that at Konakovo [38,39,40]. Advances in environmental monitoring, animal welfare, and physiological indicators are expected to support both conservation and commercial production, particularly in the context of increasingly frequent chronic heat stress [41,42,43].

## 4. Reproductive Biology of *Acipenser baerii*

### 4.1. Sexual Maturation and Gonadal Development

The Siberian sturgeon adopts a stage-based life strategy that prioritizes extensive somatic growth before energy is redirected toward reproductive metabolism [20,44]. Sexual maturity occurs relatively late, and its onset varies considerably depending on environmental temperature, food availability, growth rate, and latitude [20]. Natural populations reach sexual maturity at different ages across the species’ extensive geographic range, with females generally maturing between 11 and 22 years and males between 9 and 19 years of age. This broad range is consistent with the pronounced latitudinal variation in life history traits observed in *A. baerii*: populations inhabiting colder northern environments generally exhibit slower growth and later maturation, whereas more favorable thermal and trophic conditions can promote earlier reproductive development [20,45]. Under aquaculture conditions, however, optimized temperature regimes and continuous feeding can substantially shorten this interval, with males reaching maturity at 3–6 years and females at 6–8 years, while controlled reproduction allows gonadal development to be synchronized with production cycles [15,46,47]. Gonadal differentiation can be observed around 18 months of age, whereas molecular sexual differentiation may begin much earlier, as early as 3–6 months after hatching [48,49]. Analyses conducted on the offspring of gynogenetic females have allowed for a more precise structural characterization of this process [50].

The absence of external sexual dimorphism in *A. baerii* means that sex identification and maturity assessment, for both wild and aquacultured specimens, depend on gonadal biopsies, plasma hormone assays, and ultrasound examinations [15,46,51]. Ultrasound examination has proven particularly effective for sex determination in young specimens, offering a non-invasive alternative to biopsy [52], and the ovarian structure, including the organization of oocyte nests, has been described in detail in farmed females through histomorphological analyses [53].

In females, the progressive increase in 17β-estradiol during vitellogenesis remains one of the most reliable biochemical markers of approaching puberty [46]. In Siberian sturgeon, circulating 17β-estradiol concentrations are generally low but increase with advancing gonadal development; values ranging from approximately 0.3 to 1.2 ng/mL have been reported in females during reproductive hormonal monitoring [46,54], while a concentration of 0.35 ± 0.09 ng/mL was reported at an early stage of ovarian development [55].

However, circulating steroid concentrations may vary considerably according to gonadal stage, age, reproductive condition, and experimental or culture conditions. Testosterone concentrations may also increase toward the end of maturation, although their magnitude is strongly dependent on reproductive stage and hormonal status [46,56]. The final maturation of oocytes is controlled by progestins, particularly 17,20β-dihydroxy-4-pregnen-3-one, while spermatogenesis is controlled by androgens, specifically testosterone and 11-ketotestosterone [15,46].

Sperm morphology and biochemical changes during short-term storage provide additional markers of reproductive status [57,58]. The reproductive process is triggered by GnRH, which stimulates pituitary secretion of gonadotropins and gonadal steroidogenesis [46], and reproductive performance can be predicted by the in vitro ovulation rate of isolated follicles [59]. Dietary phytoestrogens, particularly those derived from soybean, can exhibit estrogenic activity in Siberian sturgeon and induce vitellogenin synthesis in immature individuals or males [16,18]. Although such endocrine effects do not alter the genetically determined sex, exposure to estrogenic compounds during the sensitive period of gonadal differentiation may influence phenotypic sex differentiation [60].

### 4.2. Ovarian Cycle, Fecundity, and Reproductive Ecophysiology

A defining reproductive characteristic of *A. baerii* is asynchronous gonadal development: females do not produce eggs annually, and the extended intervals between egg-laying serve to replenish energy reserves depleted between reproductive events [53,61,62]. In natural habitats, the resting interval between successive spawning events averages 3–5 years, whereas under optimized aquaculture conditions, this interval may be reduced to 1–2 years [20,63,64].

Histological analyses of farmed broodstock indicate that the biennial ovarian cycle is the most commonly observed pattern, though annual and triennial cycles have also been recorded within the same population, reflecting substantial individual variability [62]. With respect to fecundity, *A. baerii* produces eggs of intermediate diameter (3.0–3.6 mm), with individual mature oocyte mass ranging from 10.8 to 25.0 mg [20]. Despite the relatively small mass of individual oocytes, females exhibit high relative fecundity, ranging from 9 to 34 eggs/g of body weight [20]. Thus, smaller individual oocyte size does not imply low reproductive output. Rather, the production of a larger number of relatively smaller oocytes contributes to the high-relative-fecundity characteristic of *A. baerii* [20].

The gonadosomatic index (GSI) of female *A. baerii* can vary considerably with reproductive stage and environmental conditions, with reported values ranging from approximately 11% to 67% [20]. Therefore, GSI should be interpreted in relation to reproductive stage and should not be considered alone as a direct indicator of reproductive performance. In natural spawning habitats, females select hard substrates of rock or gravel in deep fluvial zones with moderate current velocity, conditions that facilitate secure egg adhesion while preventing siltation-induced asphyxiation [49,65,66].

Optimal water temperature ranges for natural reproduction and hatching vary among river systems, ranging from 8–18 °C in the Lena River to 16–21 °C in the Kolyma system. This variation does not simply follow the broader regional temperature gradient but may reflect differences in local environmental conditions and population-specific thermal adaptation [20,49].

### 4.3. Embryonic Development and Early Life Stages

Embryogenesis in *A. baerii* proceeds through well-defined sequential stages, including asymmetric holoblastic cleavage, blastulation, gastrulation, neurulation, and organogenesis, with gastrulation representing a particularly sensitive phase susceptible to developmental abnormalities [50,67,68,69]. Developmental rate is strongly temperature-dependent, with elevated incubation temperatures accelerating hatching while potentially compromising embryonic survival and modulating the yolk sac absorption rates [50,70,71]. Upon hatching, the prolarvae emerge approximately 10–12 mm in length, with their mouths still closed, relying exclusively on the endogenous reserves of the yolk sac for nutrition, a stage during which energy is produced through an intense fatty acid metabolism [15,66,67].

Hatchlings initially exhibit positive phototaxis and vertical swimming behavior (swim-up and drift), transitioning to benthic locomotion and shoaling as yolk absorption progresses [49,66]. The shift to exogenous feeding occurs approximately 9–11 days post-hatching at 18 °C and represents a critical developmental threshold for larval survival. Under experimental rearing conditions, mortality during the transition to exogenous feeding has been reported at 2.1–23.5%, corresponding to approximately 76.5–97.9% survival, with most losses occurring between 9 and 18 days post-hatching [15,46,47,70].

The introduction of live feeds such as *Artemia nauplii* at this stage significantly enhances early growth and survival rates, though gradual transition to microdiets is feasible [51,70]. Skeletal and digestive anomalies may emerge under suboptimal nutritional or environmental conditions, such as sustained elevated temperatures, during early ontogeny [30,47], while high stocking densities may reduce juvenile growth performance through competition and stress responses, despite the species’ relatively high density tolerance [51,72,73,74].

### 4.4. Artificial Reproduction and Hatchery Management

*Acipenser baerii* does not reproduce spontaneously in captivity and therefore requires controlled broodstock management incorporating both thermal conditioning and hormonal stimulation [15,46,75]. A mandatory pre-spawning wintering phase (vernalization) requires maintaining broodstock at 2–8 °C for a minimum of one to three months, a thermal cue essential for completing gametogenesis and ensuring gamete quality prior to the spring spawning induction [15,46,59,76]. Water temperature is subsequently raised progressively to 15–16 °C to initiate the ovulatory response [59,77]. Selection of reproductively competent females for hormonal induction represents the principal technical challenge in hatchery operations, as operators must prevent ovarian follicular atresia [62].

Ovulation and spermination are induced using carp pituitary extract or synthetic GnRH analogues (such as LHRHa), the latter increasingly preferred due to superior egg quality outcomes and reduced physiological stress; females typically receive two injections (a priming dose and a resolving dose), while males receive a single dose, sometimes combined with dopamine antagonists to enhance response [15,46,76,77]. Cryopreservation can substantially affect sperm quality, with reported post-thaw motility ranging from approximately 20 to 58% depending on the cryopreservation protocol [78,79]. In a recent study, sperm viability decreased from 97.9 ± 1.3% in fresh semen to 61.1 ± 3.8% and 70.0 ± 3.2% following cryopreservation with methanol and dimethyl sulfoxide, respectively [79]. Functional quality was also affected, with fertilization rates of 51.2% and 6.2% reported for methanol and dimethyl sulfoxide preserved sperm, respectively [79]. Gamete collection may be performed through minimally invasive approaches such as the Podushka technique, involving a small incision of the oviduct wall, key-hole microsurgery, which circumvents the need to sacrifice mature broodstock females, or via surgical laparotomy depending on individual size and developmental stage [15,80,81,82].

Fertilization is carried out using the semi-dry method, followed by egg de-adhesion with clay, milk, talc suspensions, tannin solutions, or diluted sodium hypochlorite, and subsequent incubation in Weiss or Osetra-type incubators at optimal temperatures of 12–16 °C for *A. baerii* [15,25,50,83]. Finally, sperm cryopreservation offers an important tool for genetic resource conservation and broodstock optimization, though sperm quality (DNA integrity, motility, oxidative stress) varies considerably among individual males and standardized protocols, such as optimal cryoprotectants like methanol or dimethyl sulfoxide (DMSO), remain an active area of development [46,78,79,82,84].

## 5. Products Derived from *Acipenser baerii*: Production Systems, Quality, and Market Positioning

### 5.1. Production Systems and Resource Efficiency

Sturgeon aquaculture relies on a mix of production technologies, and their share differs considerably among regions. Table 1 summarizes the main production systems used in sturgeon aquaculture and their reported global and regional shares. Local resource availability influences technology selection, with cages widely used in China and Russia and pond-based systems common in Western and Central Europe [3,85]. However, recent environmental regulations have begun to restrict open cage culture in inland waters, notably in China, driving a shift towards land-based systems [47]. The RAS is increasingly adopted because of its high water reuse rates and greater control over rearing conditions [51,86,87,88]. *A. baerii* is an important caviar-producing species in France, China, Italy, and Uruguay [11,89,90,91]. However, the reported 84% share attributed to China concerns total global sturgeon aquaculture production rather than global caviar production [11,89].

Stocking density also directly affects welfare and productivity. In early stage *A. baerii larvae*, densities of 30–120 larvae/L did not induce significant differences in cortisol levels, although lower densities promoted better growth [73]. In juvenile hybrid sturgeon (*A. baerii × A. schrenckii*), prolonged crowding at 200–350 fish/m^3^ for 60 days increased cortisol levels, impaired growth, and reduced antioxidant capacity [92]. Juvenile *A. baerii* can recover from nutritional stress: after periods of feed restriction followed by re-feeding, they show compensatory growth, indicating a capacity for metabolic recovery [93,94].

**Pathogens and disease burden.** Intensive farming of *A. baerii* may increase exposure to infectious agents, particularly under conditions of high stocking density or suboptimal environmental management.

Bacterial infections represent an important health concern, with *Aeromonas salmonicida* reported in juvenile *A. baerii* during a mortality outbreak and associated with hemorrhagic and ulcerative dermatitis, gill inflammation, and lesions compatible with septicemia [95]. Other *Aeromonas* spp. have also been reported as pathogenic agents in farmed Siberian sturgeon [96]. Consequently, adequate water quality, biosecurity, stocking management, and nutritional status are essential for reducing disease risk and maintaining fish health.

The main differences in rearing conditions between the wild environment and these production systems are summarized in Table 2, providing context for the welfare and stress responses discussed above.

### 5.2. Caviar Harvesting Methods and No-Kill Technology

Caviar is produced through two main methods: conventional harvesting, which sacrifices females after 6–10 years for premium malossol caviar with strong stability and shelf life [11,15], and no-kill technology, which allows five to seven harvest cycles over 15+ years per female, improving asset utilization, stabilizing output, and lowering long-term costs [11,107,108,109].

No-kill relies on ovulated eggs, whose soft, adhesive membranes require post-harvest stabilization (e.g., thermal coagulation, tannin cross-linking, patented molecular treatments, or pH-adjusted cold-water processing) to match conventional caviar’s texture and stability [63,109,110,111,112]. Extraction uses minimally invasive techniques such as the Podushka (oviduct incision), the minimally invasive surgical technique (MIST), and abdominal stripping methods [15,80,113]. Codex Alimentarius permits labeling such products as caviar when methods are declared, and strictly requires that any hormones used to induce ovulation be approved by the competent authorities, supporting market integration [11,114].

From a production and resource utilization perspective, no-kill technology may offer important advantages by allowing for the repeated use of valuable broodstock and reducing the need to replace mature females. However, these potential benefits should not be interpreted as evidence of superior animal welfare. Repeated hormonal induction, anesthesia, surgical intervention, handling, and recovery may impose welfare challenges, including physiological stress, tissue damage, infection, and mortality risks [81,108,115,116].

In addition, no-kill caviar may differ from conventionally harvested caviar in bead integrity, texture, and mouthfeel because ovulated eggs undergo changes in their membrane properties after contact with water [63,107,117,118,119].

Thus, no-kill production represents a potential sustainability and broodstock utilization strategy, but its overall advantage depends on balancing production efficiency and resource conservation against product quality and animal welfare considerations [107,108].

### 5.3. Sensory Quality and Technological Determinants

The sensory characteristics of *A. baerii* caviar are directly influenced by post-harvest processing, diet, and oocyte maturity stage [63,120]. Premium caviar is valued for its delicate buttery, nutty, and subtle oceanic aromas, complemented by a balanced texture combining slight resistance on the palate with a creamy finish, driven by free amino acids and specific nucleotides (e.g., inosinic acid) that impart a strong umami taste [121,122].

Siberian sturgeon caviar is frequently compared to *Acipenser gueldenstaedtii* (Osetra) in terms of egg size and coloration, though its sensory profile is characterized by buttery and yeasty notes with mild sweetness and earthy nuances, in contrast to the more pronounced marine or anchovy-like aromas typical of wild-caught sturgeons [63,110]. Recent advanced aroma profiling further describes the scent of both species with “fatty”, “fishy”, “seawater-like”, and “green/grassy” notes, which are primarily driven by aldehydes and alcohols derived from lipid oxidation during maturation [123].

A major technological challenge in quality control is the prevention of off-flavors, particularly the earthy or muddy taste caused by the volatile compounds geosmin and 2-methylisoborneol (MIB) produced by cyanobacteria and actinomycetes present in the rearing water, which rapidly accumulate in adipose tissue and roe [124,125]. To mitigate this defect, producers implement a pre-harvest purification protocol, in which sturgeons are kept in clean freshwater without being fed for several weeks prior to roe harvesting [125,126,127]. Additionally, seasonality and the duration of maturation heavily impact the volatile profile; for instance, spring-harvested caviar often exhibits higher levels of desirable “milky” or “buttery” flavors, while an optimal cold-storage maturation period of 6 to 9 months is critical for proper flavor development [125].

Post-harvest processing parameters also substantially influence the quality of the final product: for example, rinsing the eggs with cold water at an adjusted pH produces caviar that is darker in color, firmer, and glossier with a distinct “popping” sensation, compared to washing with hot water, which can induce a slimier texture and a stronger seaweed odor [112].

### 5.4. Market Positioning

Caviar remains a high-value luxury product, although the expansion of aquaculture has increased supply and placed downward pressure on prices [3,91,128]. *A. baerii* caviar occupies an accessible-luxury segment below premium Osetra and Beluga, while accounting for approximately 31% of global caviar production volume [11,129]. Its market positioning relative to other major caviar segments is summarized in Table 3.

### 5.5. Meat and Nutritional Composition

*Acipenser baerii* meat is highly valued for its firm texture, absence of intramuscular bones, and favorable nutritional profile [63]. It is considered a protein-rich food with high biological and nutritional value [130].

*Proximate composition.* The proximate composition of *A. baerii* meat varies with diet, fish size, and rearing conditions [130,131,132,133]. Reported values for farmed specimens, including fish reared in conventional systems and RAS, are summarized in Table 4. Comparable proximate composition data for wild-caught *A. baerii* are not available in the literature, reflecting the species’ Critically Endangered status and the resulting scarcity of specimens available for compositional analysis outside aquaculture [32]. The values reported here should therefore be interpreted as representative of farmed stock only, and not as a wild-versus-farmed comparison [63].

*Amino acid profile and protein quality*. *A. baerii* muscle contains a balanced profile of essential and non-essential amino acids, with glutamic acid, aspartic acid, lysine, and leucine among the predominant amino acids [133,134]. Representative amino acid values and protein quality indicators are presented in Table 4.

**Table 4:** Proximate composition, amino acid profile, and fatty acid composition of farmed Siberian sturgeon (*Acipenser baerii*) meat.

Composition	Parameter	Value	Reference
Proximate composition	Moisture (%)	69.89 ± 2.11	[133]
	Protein (%)	15.69 ± 0.83	[133]
	Fat (%)	12.57 ± 2.20	[133]
	Ash (%)	1.10 ± 0.08	[133]
Amino acid profile	Glutamic acid (g/100 g protein)	14.91 ± 0.26	[133]
	Aspartic acid (g/100 g protein)	10.20 ± 0.18	[133]
	Lysine (g/100 g protein)	10.02 ± 0.17	[133]
	Leucine (g/100 g protein)	7.97 ± 0.13	[133]
	Total essential amino acids (g/100 g protein)	47.62	[133]
Fatty acid profile	Total PUFA (% of total fatty acids)	32.90 ± 0.38	[135]
	EPA (20:5 n-3) (% of total fatty acids)	6.83 ± 0.13	[135]
	DHA (22:6 n-3) (% of total fatty acids)	7.95 ± 0.34	[135]
	Total n-3 fatty acids (% of total fatty acids)	20.20 ± 0.35	[135]
	Total n-6 fatty acids (% of total fatty acids)	9.99 ± 0.26	[135]
	n-3/n-6 ratio	2.04 ± 0.08	[135]

Note: Values refer to farmed *A. baerii* and are reported as mean ± SD where available. Proximate composition (moisture, protein, fat, and ash) is expressed on a wet-weight basis; amino acid values are expressed per 100 g of protein. Fatty acid percentages represent proportions of total fatty acids. Reported values may vary with rearing conditions, diet, fish size, and processing.

*Lipid profile.* Sturgeon meat is a valuable source of polyunsaturated fatty acids (PUFAs). In farmed *A. baerii*, EPA and DHA represented 6.83 ± 0.13% and 7.95 ± 0.34% of total fatty acids, respectively [133]. The complete fatty acid profile is summarized in Table 4. Fatty acid composition is influenced by dietary lipid sources, while early n-3 HUFA supplementation may affect growth, antioxidant response, and lipid metabolism in *A. baerii* [130,134,135,136].

*Color and texture*. The flesh color is species-specific, generally exhibiting achromatic tones perceived as varying shades of gray, with intermediate lightness (L*) values [63]. Instrumental analysis reveals slightly negative redness (a*) values and positive yellowness (b*) values, the latter differing significantly between Siberian and white sturgeon [63]. Color varies along the fillet: the cranial and medial zones appear whiter and more luminous, while the caudal region is darker, more reddish, and more yellowish due to higher intramuscular fat content and the presence of red muscle fibers [137]. Hybridization and fish age also influence color parameters. In Siberian × Russian sturgeon hybrids, lightness (L*) decreases and redness (a*) increases with advancing age (5 to 7 years), whereas Russian sturgeon meat maintains high lightness regardless of age [138]. Texture is firm, dense, and free of intramuscular bones, making sturgeon meat an excellent substitute for chicken breast, pork, or veal [129]. Textural parameters including hardness, gumminess, and chewiness are key determinants of consumer acceptance [139]. Hybridization can further enhance textural properties through heterosis. The Siberian × Amur sturgeon hybrid demonstrates markedly superior hardness and chewiness compared with parental species, attributed to muscle fiber hyperplasia [139].

*Sensory profile.* In fresh conditions, sturgeon meat exhibits a fresh odor with floral, fruity, or sweet notes derived from certain esters (e.g., methyl hexanoate) and alcohols [140]. The aromatic profile may also include marine, fatty, or grassy attributes produced by aldehydes (e.g., hexanal, nonanal, heptanal) and alcohols (e.g., 1-octen-3-ol) formed via enzymatic breakdown of fatty acids [141]. Off-flavor defects caused by geosmin and 2-methylisoborneol may also affect sturgeon meat, as detailed in Section 5.3.

*Microbiological safety and shelf life.* The shelf life of chilled sturgeon fillets under aerobic packaging is limited to approximately 8 days at 4 °C, after which total aerobic colony counts (ACCs) exceed the safety threshold of 7.0 log CFU/g and total volatile basic nitrogen (TVB-N) surpasses 20 mg/100 g [140]. The dominant spoilage microorganisms are *Pseudomonas fluorescens*, *P. mandelii*, *and Shewanella putrefaciens*, which metabolize proteins and lipids, generating volatile malodorous metabolites (e.g., hexanenitrile, thiazole, ammonia derivatives) and bitter-tasting hypoxanthine [141,142].

*Market forms.* Although often considered a co-product of the caviar industry, sturgeon meat is commercially vital for farm profitability and is marketed fresh, frozen, or smoked, as whole fish, steaks (medallions), or fillets [129,143]. Within the European Union, the majority of farmed sturgeon meat is frozen and exported to Eastern European and Russian markets, where strong culinary traditions support high consumer demand [63,125]. Smoked fillets command premium prices in specialized retail, while boneless meat is increasingly used in fish paste products, stuffed pastries, and raw preparations such as sashimi, sushi, and the traditional Russian stroganina [129]. The market is dominated quantitatively by males, which are slaughtered shortly after sex determination and sometimes fetch higher prices than females retained for caviar production [129].

### 5.6. Caviar. Nutritional Composition, Sensory Properties, and Food Safety

Caviar, defined as the unfertilized, salted roe of sturgeon, remains the primary commercial product and continues to dominate the market. It is colloquially known as “black gold” due to its gastronomic prestige and high economic value. Its energy value ranges from approximately 202 to 271 kcal/100 g, with the largest variation being associated primarily with differences in lipid content among sturgeon species and origins, while protein content also contributes to the overall energy value [123,144,145].

Sturgeon caviar represents a nutritionally valuable product, providing high-quality protein and lipids, as well as a broad spectrum of essential amino acids and biologically relevant fatty acids. Its proximate composition varies among species and production or processing conditions. The amino acid profile is characterized by the presence of all essential amino acids, while the lipid fraction contains substantial proportions of monounsaturated and polyunsaturated fatty acids, including the nutritionally important long-chain n-3 fatty acids EPA and DHA [63,90,145,146]. Differences in fatty acid composition have also been reported between cultured and wild-origin caviar [146]. The main reported characteristics of caviar proximate composition, amino acid profile, and fatty acid composition are summarized in Table 5.

*Sensory profile.* The sensory experience of caviar consumption involves a complex interaction between texture, taste, and aroma. Texture is a primary quality criterion: during mastication, the characteristic “popping” phenomenon occurs, whereby the egg membrane ruptures under mechanical pressure, releasing a fine, creamy, and unctuous internal content [112,121]. Post-harvest processing significantly influences textural properties: cold-processed caviar treated with pH-adjusted water exhibits superior firmness, popping intensity, and surface gloss, whereas mild thermal treatment (pasteurization) may yield a slightly stickier and less elastic texture [112,120].

Due to elevated salt and free amino acid concentrations, the primary taste profile of caviar is a harmonious combination of saltiness, mild sweetness, slight acidity, and pronounced umami [112,121]. The umami character is primarily driven by nucleotides (including inosinic acid and AMP) and glutamic acid [120,121]. In premium caviar (e.g., *Huso huso*), elevated concentrations of gamma-glutamyl peptides contribute an additional complex, round gustatory dimension known as “kokumi” [120].

The aromatic profile is largely mediated by volatile organic compounds (VOCs) derived from enzymatic degradation and oxidation of free fatty acids, including aldehydes (hexanal, nonanal, heptanal), alcohols (1-octen-3-ol), and ketones [121,122]. During cold storage, changes in bacterial communities may also contribute to alterations in the flavor profile of sturgeon caviar [147]. The optimal aromatic profile is characterized by notes of sea breeze, marine algae, oyster, butter, and hazelnut [112,120,122]. Off-flavor defects arise primarily from the accumulation of geosmin, which imparts an undesirable earthy or muddy taste the same compound responsible for sensory defects in sturgeon meat, as discussed in Section 5.5 [122].

*Contaminants and food safety considerations.* Beyond nutritional composition, contaminant exposure represents an additional food safety consideration for sturgeon products. Recent analysis of farmed *Acipenser baerii* caviar from Iran detected essential elements, including Zn, Cu, Fe, and Se, as well as potentially toxic elements such as Cd, Pb, Cr, As, and Hg [148].

Concentrations of the toxic elements were low and remained below international safety thresholds [149]. In wild-origin Eurasian sturgeon caviar, organic contaminants, including DDT, HCHs, PCBs, and PBDEs, together with multiple metals, have also been detected [150]. In addition, microplastics have been detected in the gastrointestinal tract and gills of wild *Acipenser persicus* from the Caspian Sea, demonstrating that emerging contaminants may also represent a relevant environmental concern for sturgeon [151].

Although species- and production-system-specific data for *A. baerii* remain limited, these findings highlight the importance of continued monitoring of chemical and emerging contaminants in farmed and wild sturgeon products.

*Microbiological safety and quality management.* Freshly extracted roe is nearly sterile; however, it is highly susceptible to contamination during processing due to its high water content, near-neutral pH (5.78–6.46), and rich nutrient composition [63]. The predominant specific spoilage organisms (SSOs) identified in sturgeon caviar belong to the genera *Pseudomonas* (*P. fluorescens*, *P. mandelii*), *Shewanella putrefaciens*, and *Acinetobacter* [144]. These bacteria metabolize proteins and lipids, leading to the accumulation of total volatile basic nitrogen (TVB-N), biogenic amines (histamine, cadaverine), and thiobarbituric acid reactive substances (TBARS), with concomitant deterioration of the volatile compound profile and development of rancid off-odors [123,144].

Microbial growth, including that of hazardous pathogens such as *Clostridium botulinum* and *Listeria monocytogenes*, is controlled through a combination of salting (3–6% NaCl), maintenance of water activity (aw) below 0.97, and cold-chain storage at −2 °C to +4 °C [66]. To extend shelf life and maintain total viable counts (TVCs) below the rejection threshold of 6.0 log CFU/g, producers may apply permitted preservatives such as sodium tetraborate (E285), which effectively inhibits spoilage bacteria within EU-regulated limits or mild pasteurization [63,120,144]. Natural antimicrobial agents, including nisin, lysozyme, and polyphenols, have also demonstrated efficacy in suppressing psychrophilic bacterial growth under refrigerated storage conditions [144].

### 5.7. By-Products. Collagen, Gelatin, Oils, and Bioactive Compounds

Sturgeon processing generates substantial quantities of by-products, including skin, swim bladder, viscera, and cartilage, which can be valorized to improve economic efficiency and environmental sustainability [89].

*Skin and leather.* Sturgeon skin can be tanned into high-quality leather (shagreen), used in luxury leather goods. This practice has recently been revitalized in Europe, particularly in France, as part of circular economy initiatives in aquaculture [89,106].

*Swim bladder and isinglass*. The swim bladder is the traditional raw material for isinglass, a highly purified collagen historically used as a clarifying agent in wine and beer production and in art conservation and cabinet-making due to its superior adhesive properties [30,48].

*Oils and lipids*. Oils extracted from viscera or liver are rich in unsaturated fatty acids and may be exploited for food supplements or pharmaceutical applications [130].

*Gelatin, collagen, and cartilage*. Skin and cartilaginous tissues are rich in collagen, with soluble collagen representing a significant fraction of total muscle collagen [130]. Cartilage is a natural source of chondroitin sulfate, widely used in nutraceutical formulations targeting joint health [148].

### 5.8. Applications in the Nutraceutical, Cosmetic, and Biomedical Industries

*Cosmetic applications.* Caviar extracts are widely incorporated into high-end cosmetic products, including anti-aging creams and serums, due to their rich content of proteins, lipids, vitamins, and minerals. These formulations are marketed for their moisturizing and regenerative properties, with luxury brands such as La Prairie and Dermastir using caviar as a symbolic and functional ingredient [11].

*Nutraceutical applications.* Sturgeon-derived oils are rich in the long-chain polyunsaturated fatty acids EPA and DHA, supporting their potential use as ingredients in functional foods and dietary supplements [134]. Additionally, protein hydrolysates obtained from meat or by-products may exhibit antioxidant activity and potential health-promoting properties [63].

*Biomedical applications*. Chondroitin sulfate extracted from sturgeon cartilage has demonstrated immunomodulatory effects, including increases in thymus mass and mast cell numbers in experimental animal models, indicating potential therapeutic relevance [148]. Isinglass also retains technical and biomedical relevance due to its exceptional purity and collagen structure [48].

## 6. Knowledge Gaps and Future Research Priorities

Although *Acipenser baerii* has attracted increasing scientific attention in recent decades, owing to its rapid adaptation to commercial aquaculture, and is often described as a ‘living fossil,’ substantial knowledge gaps still persist across multiple biological and applied domains. Addressing these gaps requires multidisciplinary approaches integrating molecular biology, behavioral ecology, reproductive physiology, and advanced monitoring technologies, in order to ensure both the success of conservation programs and the long-term sustainability of aquaculture production systems.

### 6.1. Sex Determination and Early Genetic Diagnostics

The genetic mechanism underlying sex determination in *A. baerii* remained unclear for a long time. Although a WZ-type model had been proposed, until recently no major gene had been identified, nor were there any reliable molecular markers for early diagnosis [60]. The prolonged rearing of males, which do not produce caviar, generates additional costs estimated at up to 30% of rearing expenses [47,152], and current methods, ultrasound and endoscopy, become effective only after 3–5 years [101,153].

The research priority was early sex determination (ESD) through the identification of molecular markers during the window of sexual differentiation [60]. Implementing ESD at the juvenile stage (~0.5 years) offers a clear economic advantage, allowing for the early separation of males and the optimization of rearing space [153]. However, the development of reliable PCR markers, such as AllWSex2, has been hampered by the genomic complexity of sturgeons, which retain an evolutionary octoploid karyotype of ~250 chromosomes and sex chromosomes that are poorly differentiated microscopically [40,75,154].

Despite these structural challenges, a major recent discovery identified a female-specific genomic region dating back 180 million years, confirming the conserved ZZ/ZW sex determination system in *Acipenseridae* [154]. As a result, the AllWSex2 marker has been validated as an effective tool for qualitative PCR-based genetic sex determination, enabling high-accuracy sex identification well before any morphological gonadal differentiation [61,154].

### 6.2. Early-Life Behavioral Ecology

The behavior of prolarvae and juveniles in the wild remains poorly understood, particularly their substrate and feeding preferences [44]. Post-release survival is affected by maladaptive behaviors acquired in hatcheries; surface feeding accustoms sturgeons, which are naturally benthic, to swimming in the upper water column, increasing the risk of predation [155].

Research focuses on “survival training” prior to release: exposure to chemical signals from predators triggers avoidance reflexes [156,157], and enriching the environment with sand or gravel reduces stress and promotes natural benthic behavior [49,158,159]. Laboratory tests provide useful data, but extrapolating these findings to natural environments remains difficult [49].

### 6.3. Reproductive Physiology and Endocrine Disruption

The mechanisms that regulate sexual maturity and ovarian cyclicity remain essential for the management of breeding stock [60,62]. The hypothalamic–pituitary–gonadal axis is sensitive to contaminants: nitrates in RAS abnormally increase sex steroid levels in females, disrupting oogenesis [160,161], and thermal stress reduces gonadotropin gene expression and vitellogenin synthesis, potentially inhibiting gonadal development [162].

### 6.4. Cryopreservation and Genetic Resource Banking

Standardizing cryopreservation protocols remains a priority for genetic conservation [57]. Methanol, used as a cryoprotectant in place of DMSO, has improved post-thaw motility and fertilization of sperm, but the cryopreservation of oocytes and embryos remains unfeasible due to the large size of the oocytes, and variability in sperm quality limits the reliability of cryopreserved material [57].

Promising approaches include the cryopreservation of somatic cells via nuclear transfer [163] and the transplantation of primordial germ cells into surrogate species, such as the cega [164,165,166].

### 6.5. Environmental Monitoring and Non-Invasive Assessment Technologies

The need to assess sturgeon populations and products without sacrificing the fish has driven the development of non-invasive monitoring technologies. Acoustic telemetry (VEMCO Positioning System) allows for tracking migration routes and behavioral responses to hydroelectric flow regulation [167,168], and it is essential to assess the impact of these implanted microtransmitters on the physiology and swimming ability of juveniles [169]. Furthermore, the analysis of stable isotopes (oxygen, hydrogen, carbon, nitrogen, along with sulfur and strontium) complements genetic markers in distinguishing farmed caviar or meat from poached ones [34,75,170,171].

For quality control in aquaculture, the analysis of volatile organic compounds (SIFT-MS, GC-IMS) provides a rapid and non-destructive assessment of caviar freshness, including the early detection of flavor defects and dynamic quality assessment during cold storage [124,140,141].

Climate change vulnerability. Climate change may increasingly affect wild populations of *Acipenser baerii* through rising water temperatures and more frequent thermal extremes. Experimental studies have shown that elevated temperatures induce physiological and molecular stress responses in *A. baerii*, including increased cortisol levels, oxidative stress, activation of heat-shock proteins, inflammatory responses, and tissue damage [43,71,150].

Prolonged heat exposure may also affect telomere dynamics, with chronic exposure to 30 °C resulting in a significant reduction in telomere length in *A. baerii* [172]. However, species-specific projections of future habitat suitability for remaining wild *A. baerii* populations under climate change scenarios remain limited, highlighting the need for further research integrating climate projections, river temperature regimes, and population distribution.

## 7. Sustainability and Future Directions

**Figure 2:**
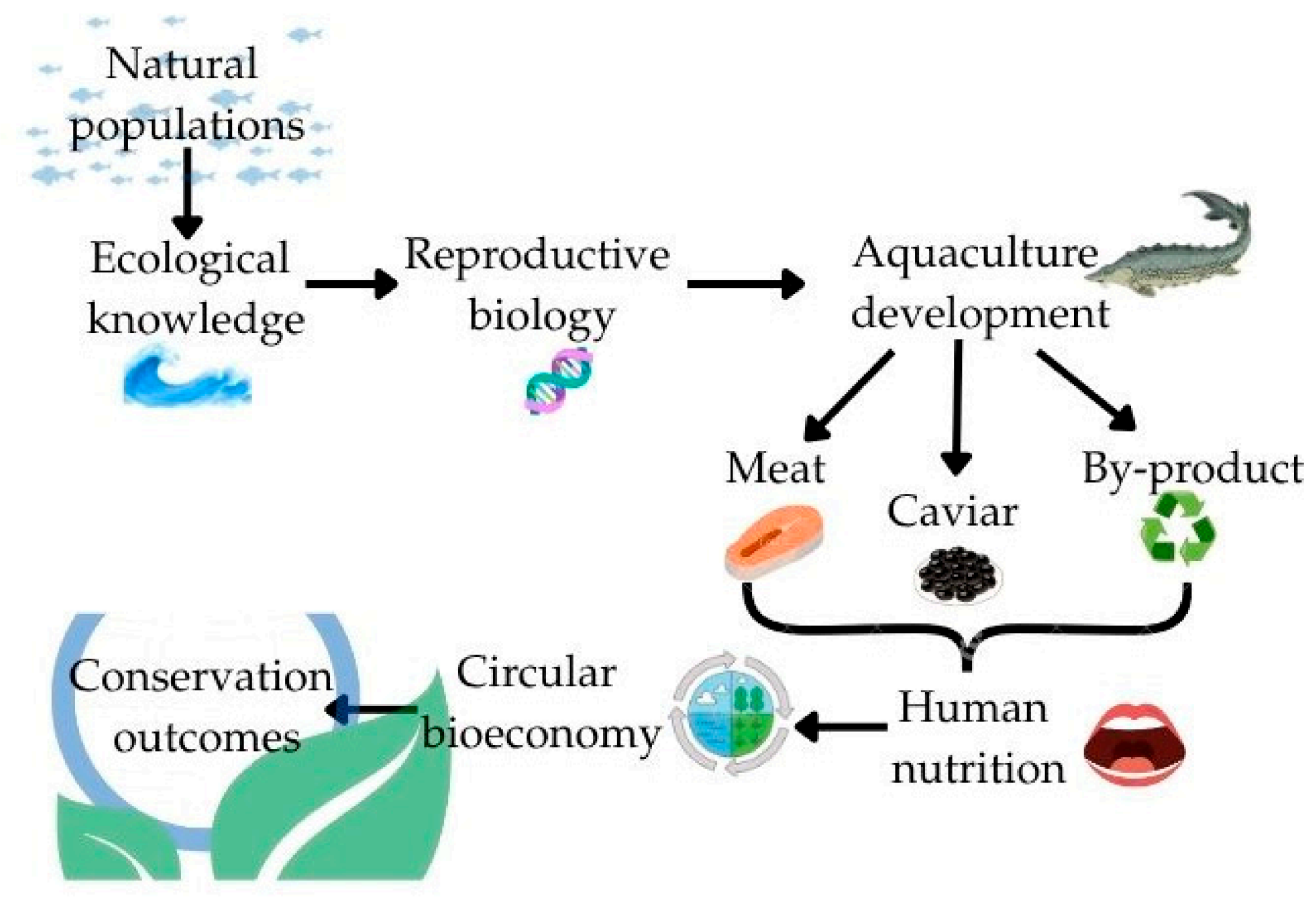
Future directions for the sustainable management and valorization of *A. baerii* resources.

Despite the significant advances achieved in the understanding and cultivation of *Acipenser baerii*, several scientific and technological challenges remain to be addressed. Future research should focus on improving genetic tools for sex determination, optimizing cryopreservation protocols, developing sustainable feed formulations, enhancing animal welfare in no-kill caviar production systems, and increasing the valorization of sturgeon-derived by-products. Drawing on the evidence synthesized throughout this review, a conceptual framework is proposed to illustrate the interconnected pathways linking the natural ecology of *A. baerii*, aquaculture development, product valorization, and conservation outcomes (Figure 2).

As illustrated in Figure 2, the sustainable management of *A. baerii* requires an integrated approach that connects ecological knowledge, reproductive management, aquaculture innovation, food production, and conservation strategies. Such a framework may support both the long-term viability of the species and the economic sustainability of the sturgeon industry.

### 7.1. Environmental Impact and Life Cycle Assessment

Long production cycles and intensive use of resources result in a significant ecological footprint for sturgeon aquaculture, which is higher than that of other fish farming systems [173]. LCA studies indicate a global warming potential of 53.4–82.9 kg CO_2_ equivalent per kilogram of caviar, depending on the species and the length of the growth cycle to maturity [90], with feed often accounting for over 70% of total emissions [91,174,175]. Strategies such as early sex determination (ESD) or the transition to renewable energy can reduce the impact by 3.7–21% [91].

Economic allocation of the impact through the utilization of meat and by-products reduces caviar’s carbon footprint by 6–14%, aligning the industry with the principles of the circular economy [91,176,177]. There remains a need to develop new protein conversion efficiency indicators tailored to long-cycle species, given the increasingly acute competition between feed and food [178] at the production system level [63,91].

### 7.2. Animal Welfare and Ethical Considerations

The principles of the “Five Freedoms” are increasingly considered in sturgeon aquaculture as well [46], and the slaughter of females after several years of rearing raises ethical concerns regarding the use of animals for caviar production [108]. No-kill technologies, including abdominal stripping and minimally invasive surgical approaches, may reduce the need for repeated replacement of mature females and therefore offer potential resource utilization and sustainability benefits. However, these benefits do not necessarily translate into improved animal welfare. Repeated anesthesia, hormonal induction, invasive handling, surgical procedures, and recovery periods may expose broodstock to cumulative physiological and handling stress [108,179,180].

Accordingly, the welfare implications of no-kill production should be assessed across the entire production cycle rather than solely on the basis of female survival. Factors such as the invasiveness and frequency of procedures, anesthesia, fasting, postoperative recovery, tissue damage, infection, feeding behavior, and mortality should be considered when evaluating welfare outcomes [78,81,108,179,180,181]. Thus, no-kill production should not automatically be regarded as an inherently more ethical alternative to conventional harvesting; rather, its welfare advantage depends on how procedures are performed and on the cumulative effects of repeated interventions. Conventional harvesting, in contrast, involves the slaughter of mature females but avoids the repeated surgical and recovery procedures associated with multiple no-kill harvest cycles. Humane slaughter therefore remains an important component of welfare assessment. Electrical stunning, percussive stunning, and ice-slurry methods have been proposed or used in fish slaughter, although their suitability and effectiveness must be evaluated according to species-specific physiological responses and validated indicators of unconsciousness [182,183,184]. The absence of fully standardized, scientifically validated welfare indicators for sturgeon and variation in slaughter practices among farms remain important challenges for ensuring consistently humane treatment [184].

### 7.3. Balancing Conservation and Market Demand

The listing of *Acipenseridae* species in the CITES Appendices has shifted commercial supply toward aquaculture, but the fraudulent substitution of cheaper roe from over 38 non-sturgeon species as genuine caviar remains a persistent problem in a competitive global market [64,123,185]. Advanced molecular methods, such as DNA barcoding (including COIBar-RFLP) and stable isotope analysis, enable the differentiation of wild caviar from farmed caviar and the rapid detection of fraudulently traded batches [34,75,127]. The expansion of the middle class and “masstige” marketing strategies have led to a significant increase in consumer demand for organic certification, animal welfare, and supply chain transparency [11,107].

## 8. Conclusions

*Acipenser baerii* (Brandt, 1869) has undergone a fundamental transition from a heavily exploited wild resource to a species whose commercial viability depends predominantly on aquaculture. The severe decline in natural populations in Siberian basins has rendered wild harvesting insufficient, and current caviar production is now derived almost entirely from aquaculture. China, Russia, and several European countries have established themselves as leading producers, supported by the species’ remarkable ecological plasticity. Being predominantly freshwater and potamodromous, *A. baerii* avoids obligatory marine–freshwater transitions, reducing the physiological demands associated with salinity changes and facilitating its culture in freshwater systems.

The sector’s sustainability depends on several interconnected factors, including the identification of viable alternative protein sources, effective genetic risk management, and prevention of accidental escapes. Escaped *A. baerii* may reproduce and hybridize with native sturgeon species, creating risks of genetic introgression and loss of genetic integrity, particularly in European river systems. Overall, the future development of *A. baerii* aquaculture, including in Romania, will depend on balancing production efficiency with environmental protection, genetic conservation, and ethical standards.

## Figures and Tables

**Table 1:** Main production systems used in sturgeon aquaculture and their reported global and regional shares.

Production System/Region	Global Share of Production Systems (%)	Regional Share/Reported Use	References
Flow-through systems (FT)	36	Widely used globally	[3,85]
Recirculating aquaculture systems (RAS)	21	Increasingly used worldwide; particularly relevant in water-limited regions	[3,85]
Cages	18	≈25% in China; up to 65% in Russia	[3,85]
Mixed FT/RAS	11	Used in several producing regions	[3,85]
Ponds	6	Widely used in Western and Central Europe	[3,85]
Other combined systems	8	FT/ponds: 4.5%; cage/ponds: 3.2%; RAS/ponds: 0.1%	[85]
Commercial sturgeon farms by region	—	China: 54%; Russia: 24%; Middle East: 8%; Far East: 7%; Europe: 6%	[85]

**Table 2:** Comparative overview of rearing conditions for *Acipenser baerii* across the wild environment and principal aquaculture production systems.

Parameter	Wild Environment	Extensive (Ponds)	Semi-Intensive	Intensive/Superintensive (RAS)	References
Space & stocking density	Vast rivers and lakes; unrestricted movement	Low density; large space; unrestricted movement within pond	Moderate density; earthen ponds with daily water exchange (10–15%)	Very high density: 500 ind./m^2^ (5–50 g); 50 ind./m^2^ (500–2000 g); biomass > 80–100 kg/m^3^.	[86,97,98,99]
Water type & temperature	Cold, flowing freshwater; seasonal variation between 0–25 °C	Natural conditions; dependent on climate and precipitation; 14–22 °C	Earthen ponds; partial water renewal; semi-controlled; 20–23 °C	Fully recirculated; strictly controlled at 18–20 °C year-round	[29,98,99,100,101]
Water quality control	Natural self-purification; dilution by river flow	Minimal; regulated by pond ecosystem	Partial; daily water exchange for refreshment	Absolute: mechanical filtration, biofiltration, UV sterilization, oxygenation; NH_3_ < 0.01 mg/L; NO_2_^−^ < 0.1 mg/L; DO > 8–9 mg/L; pH 7.0–7.5	[29,102]
Diet	Benthic macrofauna: chironomid larvae, oligochaetes, mollusks, crustaceans, small fish	Natural prey (plankton, benthic invertebrates); occasional supplemental feed	Natural food supplemented regularly with commercial pellets	Exclusive reliance on high-performance formulated pellets (fishmeal/fish oil or alternative ingredients)	[22,103,104]
Growth rate & sexual maturation	Slow growth; sexual maturity at 9–22 years depending on population	Slow to moderate growth; maturation timeline close to wild	Moderate growth acceleration	Accelerated growth: ~700 g in 14 months; sexual maturity at 3–8 years under optimal conditions	[8,50,105,106]
Disease risk & stress	Low due to low density and natural immune stimulation	Low; natural environment buffers stress	Moderate	High chronic stress due to confinement; high susceptibility to bacterial and viral infections; system failure can cause total mortality within hours	[29,72,96]
Energy & operational costs	Not applicable	Low; minimal infrastructure	Moderate	High energy consumption; complex infrastructure; high biosecurity requirements	[29]
Production output	Dependent on natural population dynamics; unpredictable	Low; slow production cycle	Moderate	Maximum yield; predictable, year-round production	[29,50,86]

RAS: recirculating aquaculture system; DO: dissolved oxygen; NH_3_: ammonia; NO_2_^−^: nitrite.

**Table 3:** Comparative market positioning of *Acipenser baerii* caviar and other major caviar segments.

Caviar Segment	Main Species/Type	Relative Price Positioning	Global Production Volume Share (%)	Reference
**Siberian caviar**	*Acipenser baerii*	Accessible luxury	31	[11,128]
**Osetra caviar**	*Acipenser gueldenstaedtii*	Premium	20	[11,128]
**Kaluga-type caviar**	*Huso dauricus* × *Acipenser schrenckii*	Premium	13	[11]
**White sturgeon caviar**	*Acipenser transmontanus*	Premium	12	[11]
**Other species and hybrids**	Various	Variable	24	[11]
**Beluga caviar**	*Huso huso*	Ultra-premium	Not reported	[128]

Note: Global shares refer to reported shares of total caviar production volume and should not be interpreted as market-value shares. Relative price positioning is indicative and may vary depending on origin, egg quality, processing, and brand.

**Table 5:** Proximate composition, amino acid profile, and fatty acid composition of sturgeon caviar reported in the literature.

Parameter	Reported Value/Characteristic	Basis of Expression	Reference
***Proximate composition***			
**Moisture**	47.72–59.5%	Wet basis	[63,90]
**Crude protein**	23.8–25.55%	Wet basis	[63,90]
**Crude lipid**	14.23–19.7%	Wet basis	[63,90]
**Ash**	1.9–3.9%	Wet basis	[63,90]
***Amino acid profile***			
**Total amino acids (TAA)**	50.31–53.54%	Dry basis	[90]
**Essential amino acids (EAA)**	37.44–38.04% of TAA	Dry basis	[90]
**Glutamic acid**	7.29–7.69%	Dry basis	[90]
**Essential amino acid score**	>1 for all essential amino acids	FAO/WHO reference pattern	[90]
***Fatty acid profile***			
**Saturated fatty acids (SFA)**	24.74–25.44%	% of total fatty acids	[90]
**Monounsaturated fatty acids (MUFA)**	38.93–43.29%	% of total fatty acids	[90]
**Polyunsaturated fatty acids (PUFA)**	29.52–35.61%	% of total fatty acids	[90]
**EPA (C20:5n-3)**	3.46%	% of total fatty acids	[145]
**DHA (C22:6n-3)**	11.2%	% of total fatty acids	[145]

Note: Values are reported specifically for *Acipenser baerii* caviar produced under aquaculture conditions in Ukraine. Values represent ranges reported in the cited studies and should not be interpreted as a standardized composition because rearing conditions and processing may influence caviar composition. Proximate composition is expressed on a wet basis, amino acid composition on a dry basis, and fatty acid composition as a percentage of total fatty acids [63,90,145,146].

## Data Availability

No new data were created or analyzed in this study. Data sharing is not applicable to this article.
